# The Repairing of the Recto-Neovaginal Fistula in a Male-to-Female Transgender Through Perineal Graciloplasty

**DOI:** 10.7759/cureus.15784

**Published:** 2021-06-20

**Authors:** Nail Omarov, Sedat Tatar

**Affiliations:** 1 General Surgery, Koç University Hospital, Istanbul, TUR; 2 Plastic Surgery, Koç University Hospital, Istanbul, TUR

**Keywords:** transgender surgery, recto-neovaginal fistula, rar (recto anal repair), perineal graciloplasty, plastic surgergy

## Abstract

Rectovaginal fistulas, which are abnormal epithelial-lined connections between the rectum and vagina, are challenging to treat. Treatment of recto-neovaginal fistulas is more complicated due to the altered perineal anatomy in individuals undergoing gender reassignment surgery. We present a recto-neovaginal fistula that occurred after reassignment surgery male-to-female transgender case of a was successfully treated with restorative perineal graciloplasty.

## Introduction

About 0.6% of the United States population identify themselves as transgender individuals [1]. The rate of the transgender population is similar in Europe [2]. In recent years, medical and surgical expenditures of these individuals, particularly for gender reassignment surgery, have been covered by the insurance companies in Europe [3,4]. However, as the number of gender reassignment surgery increases, complications including recto-neovaginal fistulas have been more frequently reported.

Rectovaginal fistulas, which are abnormal epithelial-lined connections between the rectum and vagina, are challenging to treat. Treatment of recto-neovaginal fistulas is more complicated due to the altered perineal anatomy in individuals undergoing gender reassignment surgery [5]. In this article, we present a male-to-female transgender case of a recto-neovaginal fistula which was successfully treated with restorative perineal graciloplasty.

## Case presentation

A 24-year-old male-to-female transgender adult with a body mass index of 25.8 kg/m2 who underwent gender reassignment surgery through penoscrotal flap vaginoplasty [6] in an external center was admitted to our clinic with the complaint of fecal incontinence through the vagina. Her medical history revealed no comorbidities or previous surgery, and she was using psychiatric drugs. After the initial surgery, neovaginal dilatation was performed using specifically designed dilators to prevent neovaginal stenosis, and fecal incontinence through the vagina occurred three months after the initial surgery. The patient was followed for a couple of months; however, no spontaneous closure of the fistula was noted. In our clinic, physical examination was performed in the lithotomy position under the supervision of a gynecologist, and methylene blue dye was given via the transanal route, which was fistulized to the vagina through the anterior wall. The fecal diversion was decided to prevent fecal contamination of the fistula tract and to provide secondary healing. Transverse end colostomy was performed. After three months of surgery, the patient was reexamined in the lithotomy position. However, the methylene blue dye, which was given via the transanal route was found to be fistulized to the vagina through the anterior wall. As a result, restorative perineal graciloplasty was planned. Written informed consent was obtained from the patient.

**Figure 1:**
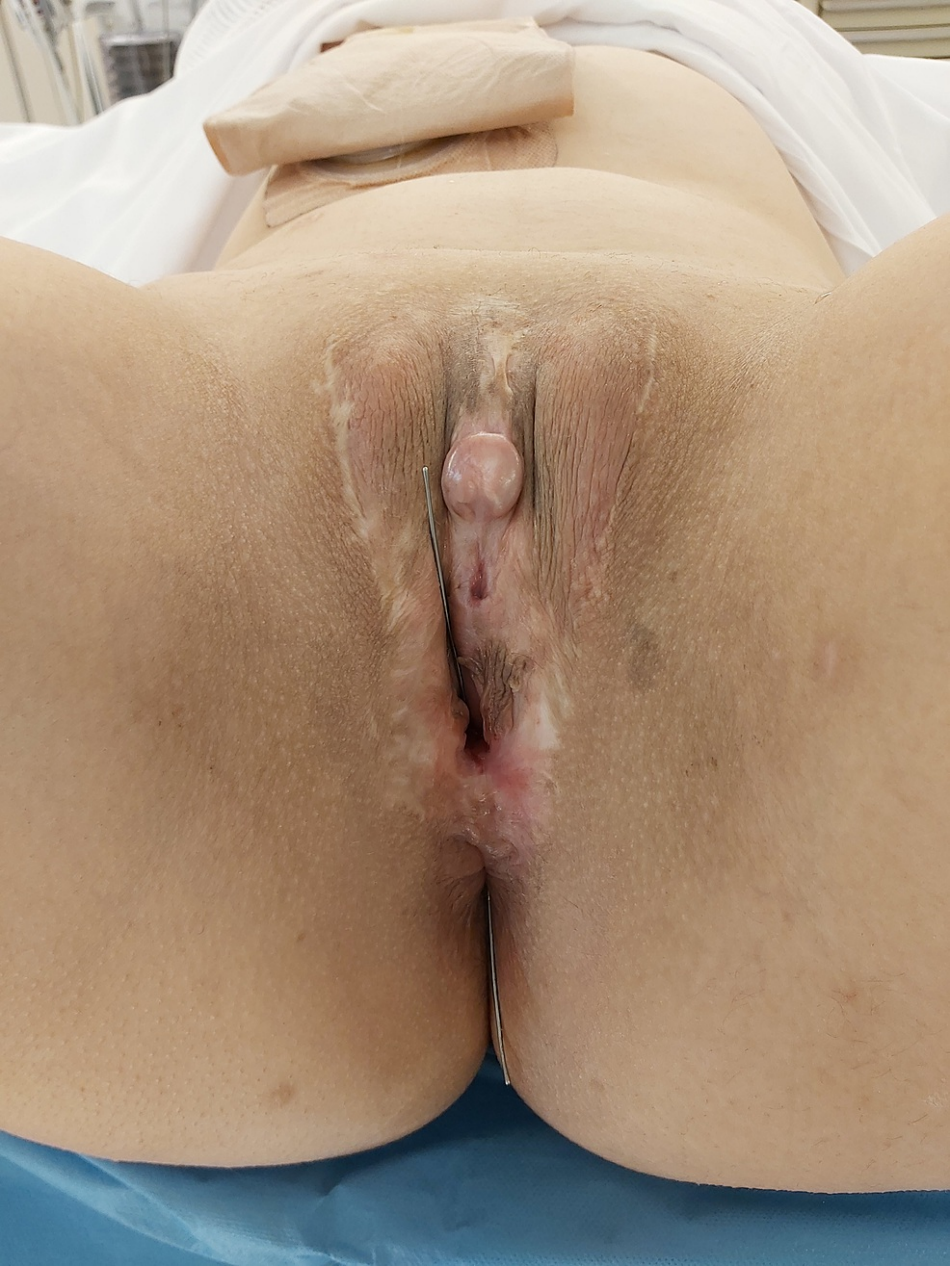
The lithotomy position and seems of the recto-neovaginal fistula.

The gracilis muscle is a long and slender muscle located in the adductor compartment of the thigh. Its transposition is a viable option for repairing fistulas between the neovagina and rectum [7]. The operation was performed under general anesthesia in the lithotomy position (Figure 1).

**Figure 2:**
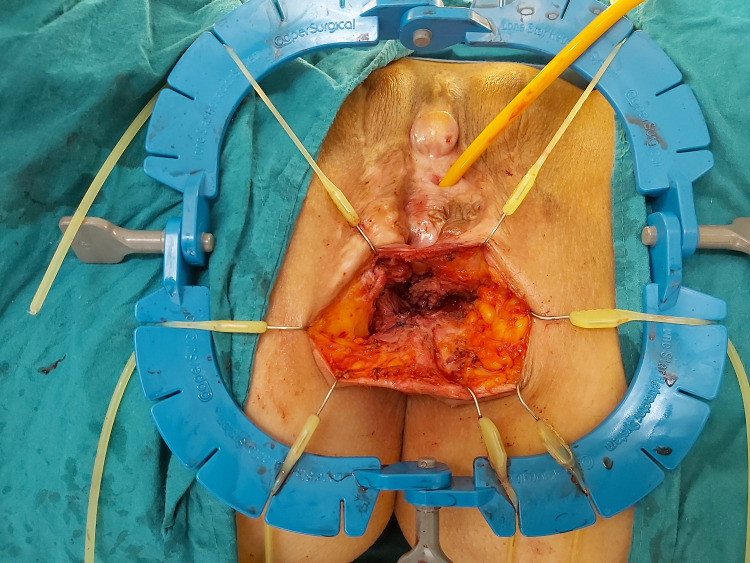
The dissection and primarily repairing of the recto- neovaginal fistula

Preoperatively prophylactic intravenous antibiotherapy was administered. The neovagina and rectum were cleaned with povidone-iodine, and a Foley bladder catheter was inserted for urine output monitoring. An anterior perineal incision was made between the neovagina and anus. The groove located between the rectum and neovagina was gently dissected, and the fistula was reached. The fistula tract was dissected and primarily repaired using tension-free Vicryl 3/0 sutures (Figure 2). After repair, hydrogen peroxide was injected into the fistula through the anus to ensure that there was no leak from the anterior wall. 

**Figure 3:**
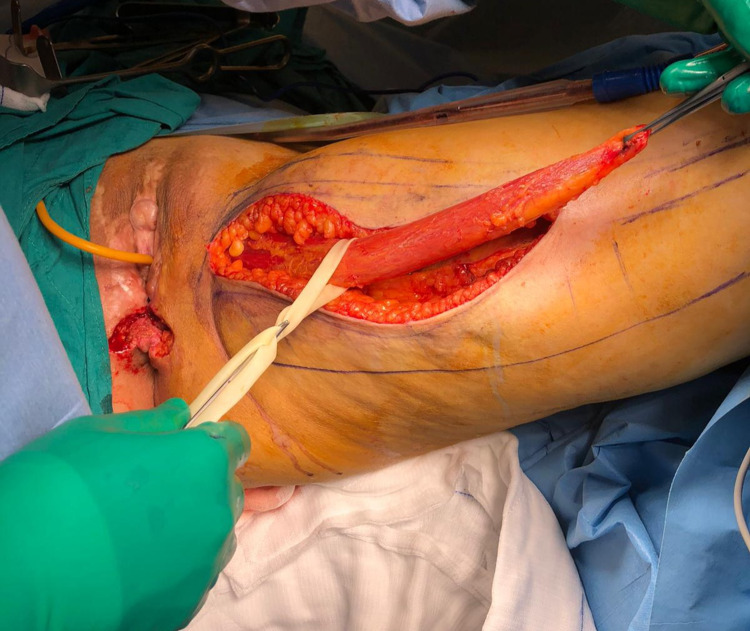
The cutting of the distal tendon of the gracilis muscle and the mobilizing with preserving the proximal neurovascular pedicle

A transverse incision from the proximal and an oblique incision from the distal left thigh was made, and the gracilis muscle was reached. Through the distal incision, the distal tendon of the gracilis muscle was cut, and the muscle was mobilized, preserving the proximal neurovascular pedicle (Figure 3).

**Figure 4:**
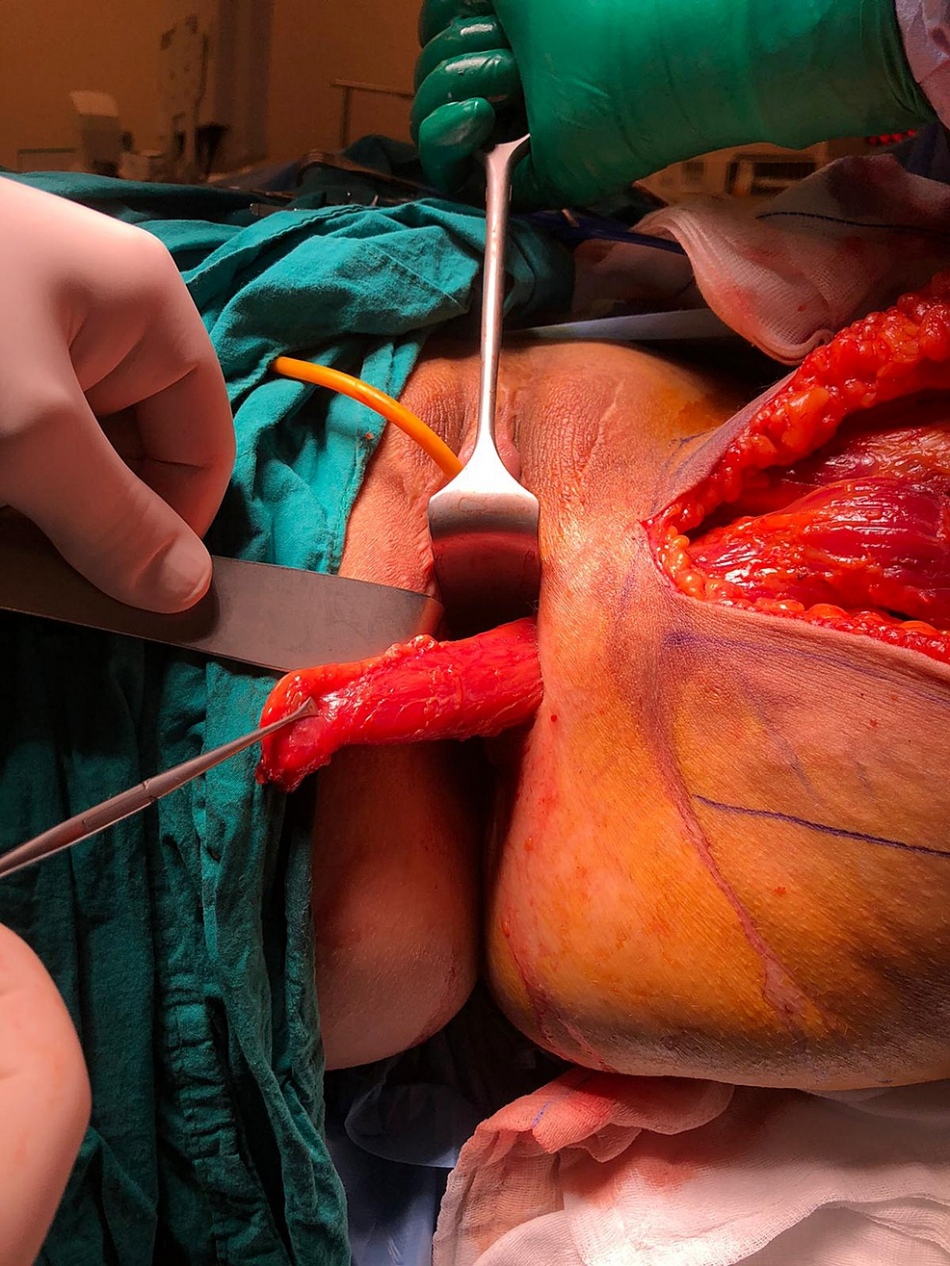
The interposition of the gracilis muscle through the subcutaneous tunnel to the between neovagina and rectum

A subcutaneous tunnel was created between the proximal thigh incision and perineal incision, and the gracilis muscle was retrieved to the perineum. The muscle was then fixed using the vicryl 3/0 sutures between the anterior rectal wall, which was repaired, and the neovagina (Figure 4).

**Figure 5:**
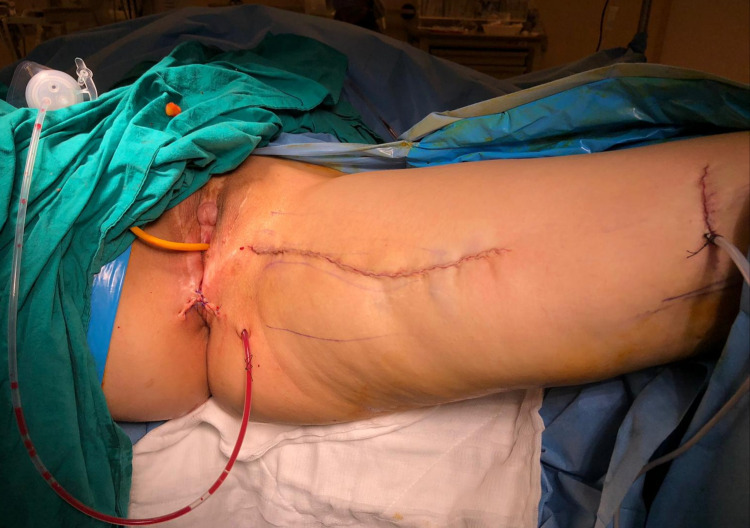
The appearance of the incisions and aspiration drainage on the surgical site

Perineal and thigh wounds were primarily repaired, and aspiration drainage was placed to the surgical site (Figure 5).

The patient was hospitalized with bed rest in the ward for two days. No postoperative complications were observed, and she was discharged on day eight. During follow-up, neovaginal dilatation was recommended to prevent neovaginal stenosis. At three months of follow-up in the outpatient setting, an examination was carried out in the lithotomy position, and methylene blue dye was given via the transanal route. No vaginal leak was seen, and the fistula was found to be completely closed. 

After three months, transverse end colostomy was successfully closed, and the patient was discharged from the hospital uneventfully on day five. At six months of follow-up, she was doing well with a healthy quality of life.

## Discussion

Male-to-female gender reassignment surgery is an extremely challenging type of surgical procedure, which may lead to rectal and bladder injuries due to their approximate and complex anatomical connection. These types of injuries restrict the use of neovagina during sexual intercourse. Surgery-related complications usually occur within the first four months [8]. Although recto-neovaginal fistulas are rare entities, they adversely affect the quality of life, sexual life, and psychological state of transgender individuals. In a cohort study, Gaither et al. [9] reported that the rate of recto-neovaginal fistulas was 0.9% in male-to-female transgender individuals. In a review, this rate was reported as varying from 0.8 to 17% [9]. In a French study, only one of 63 patients developed recto-neovaginal fistula after male-to-female gender reassignment surgery [10]. These complications usually occur during bladder and prostate dissection. Goddard et al. [11] recommended rectoscopic examination after bladder and prostate mobilization. Due to the complexity of these surgeries, they should be performed in experienced centers and the patients should be meticulously followed by experienced surgeons for postoperative complications.

Gracilis muscle transposition was first described by Garlock [12] in 1928 for the management of rectovaginal and vesicovaginal fistulas. Although it was earlier recommended to use gluteus, sartorius, and rectus abdominus muscles in the treatment of rectovaginal fistulas, these approaches were abandoned later due to low success rates, and the use of gracilis muscle was advocated by many surgical teams [13]. Of note, as creation of a colostomy for the treatment of recto-neovaginal fistulas often remains inadequate, additional procedures are required. Recurrent fistulas also restrict the use of neovagina during sexual intercourse. Some complicated fistulas may lead to living with a colostomy bag during the lifetime, impairing the psychological and social well-being of the individual. In our patient, we used the gracilis muscle interposition, although the literature is still scarce regarding the most optimal technique. We believe that this technique is effective and safe with a minimal complication rate.

## Conclusions

In conclusion, recto-neovaginal fistulas are one of the complications of gender reassignment surgery and difficult to treat. However, they can be successfully treated using the restorative garciloplasty technique in experienced centers.
